# 1-(Prop-2-en-1-yl)-3-{[3-(pyridin-4-yl)-4,5-di­hydro­isoxazol-5-yl]meth­yl}-1*H*-anthra[1,2-*d*]imidazole-2,6,11(3*H*)-trione

**DOI:** 10.1107/S160053681301369X

**Published:** 2013-05-22

**Authors:** Zahra Afrakssou, Youssef Kandri Rodia, Frédéric Capet, El Mokhtar Essassi, Christian Rolando, Lahcen El Ammari

**Affiliations:** a Laboratoire de Chimie Organique Appliquée, Université Sidi Mohamed, Ben Abdallah, Faculté des Sciences et Techniques, Route d’Immouzzer, BP 2202 Fès, Morocco; b Unité de Catalyse et de Chimie du Solide (UCCS), UMR 8181 Ecole Nationale Supérieure de Chimie de Lille, France; c Laboratoire de Chimie Organique Hétérocyclique, URAC 21, Pôle de Compétences Pharmacochimie, Université Mohammed V-Agdal, BP 1014, Avenue Ibn Batouta, Rabat , Morocco; d USR 3290 Miniaturisation pour l’Analyse, la Synthèse et la Protéomique, 59655 Villeneuve d’Ascq Cedex, Université Lille 1, France; e Laboratoire de Chimie du Solide Appliquée, Faculté des Sciences, Université Mohammed V-Agdal, Avenue Ibn Battouta, BP 1014, Rabat, Morocco

## Abstract

The fused five- and three six-membered rings of the anthra[1,2-*d*]imidazole­trione part of the title compound, C_27_H_20_N_4_O_4_, show two different substituents at the imidazole N atoms, *viz.* an allyl group and a [3-(pyridin-4-yl)-4,5-di­hydro­isoxazol-5-yl]methyl group. The fused-ring system is approximately planar [r.m.s. deviation = 0.232 (2) Å], but is slightly buckled along the common edge of the two pairs of adjacent rings, with a dihedral angle between them of 11.17 (6)°. The isoxazole ring makes dihedral angles of 27.2 (2) and 12.7 (2)° with the imidazole and pyridine rings, respectively. Weak C—H⋯O and C—H⋯N hydrogen bonds ensure the cohesion of the crystal structure, forming a three-dimensional network.

## Related literature

For the use of anthra­quinone as an organic redox mediator, see: Campos-Martin *et al.* (2006 ▶); Harish *et al.* (2009 ▶); Jürmann *et al.* (2007 ▶); Manisankar & Gomathi (2005 ▶). For the biological activity of anthra­quinone derivatives, see: Henderson *et al.* (1998 ▶); Barasch *et al.* (1999 ▶); Dou *et al.* (2009 ▶). For background to pH sensor applications, see: Wong *et al.* (2004 ▶); Lafitte *et al.* (2008 ▶); Wildgoose *et al.* (2003 ▶). For similar compounds, see: Afrakssou *et al.* (2010 ▶, 2011 ▶).
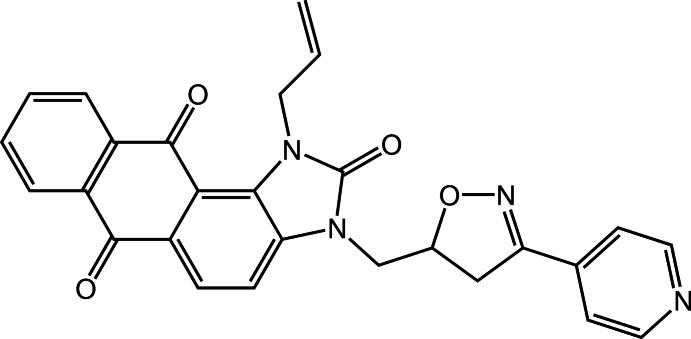


## Experimental

### 

#### Crystal data

C_27_H_20_N_4_O_4_
*M* *_r_* = 464.47
Triclinic, 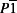

*a* = 8.0930 (2) Å
*b* = 12.1191 (3) Å
*c* = 12.2743 (2) Å
α = 87.109 (1)°
β = 73.612 (1)°
γ = 72.283 (1)°
*V* = 1099.35 (4) Å^3^
*Z* = 2
Mo *K*α radiation
μ = 0.10 mm^−1^
*T* = 296 K
0.14 × 0.10 × 0.08 mm

#### Data collection

Bruker APEXII CCD diffractometer
36799 measured reflections
5659 independent reflections
3900 reflections with *I* > 2σ(*I*)
*R*_int_ = 0.031

#### Refinement

*R*[*F* ^2^ > 2σ(*F* ^2^)] = 0.048
*wR*(*F* ^2^) = 0.148
*S* = 1.02
5659 reflections
316 parameters
H-atom parameters constrained
Δρ_max_ = 0.37 e Å^−3^
Δρ_min_ = −0.20 e Å^−3^

### 

Data collection: *APEX2* (Bruker, 2009 ▶); cell refinement: *SAINT-Plus* (Bruker, 2009 ▶); data reduction: *SAINT-Plus*; program(s) used to solve structure: *SHELXS97* (Sheldrick, 2008 ▶); program(s) used to refine structure: *SHELXL97* (Sheldrick, 2008 ▶); molecular graphics: *ORTEP-3 for Windows* (Farrugia, 2012 ▶); software used to prepare material for publication: *PLATON* (Spek, 2009 ▶) and *publCIF* (Westrip, 2010 ▶).

## Supplementary Material

Crystal structure: contains datablock(s) I, global. DOI: 10.1107/S160053681301369X/im2433sup1.cif

Structure factors: contains datablock(s) I. DOI: 10.1107/S160053681301369X/im2433Isup2.hkl

Supplementary material file. DOI: 10.1107/S160053681301369X/im2433Isup3.cml

Additional supplementary materials:  crystallographic information; 3D view; checkCIF report

## Figures and Tables

**Table 1:** Hydrogen-bond geometry (Å, °)

*D*—H⋯*A*	*D*—H	H⋯*A*	*D*⋯*A*	*D*—H⋯*A*
C3—H3⋯N3^i^	0.93	2.58	3.471 (2)	160
C3—H3⋯O4^i^	0.93	2.67	3.470 (2)	145
C19—H19*A*⋯O3^ii^	0.97	2.56	3.3356 (19)	137
C21—H21*A*⋯O3^ii^	0.97	2.45	3.350 (2)	154

Symmetry codes: (i) 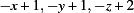; (ii) 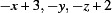.

**Fig. 1.**
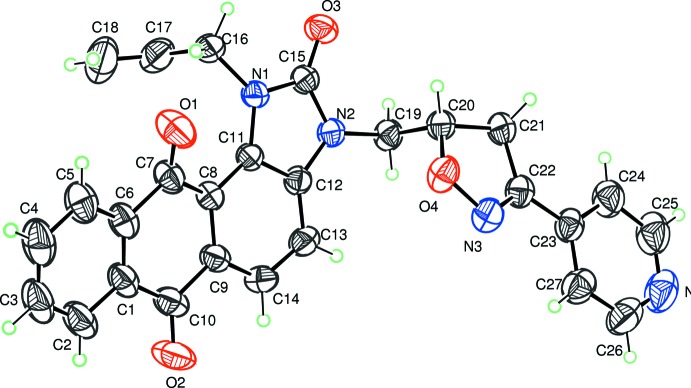
: Molecular structure of the title compound with the atom-labelling scheme. Displacement ellipsoids are drawn at the 50% probability level. H atoms are represented as small circles.

**Fig. 2.**
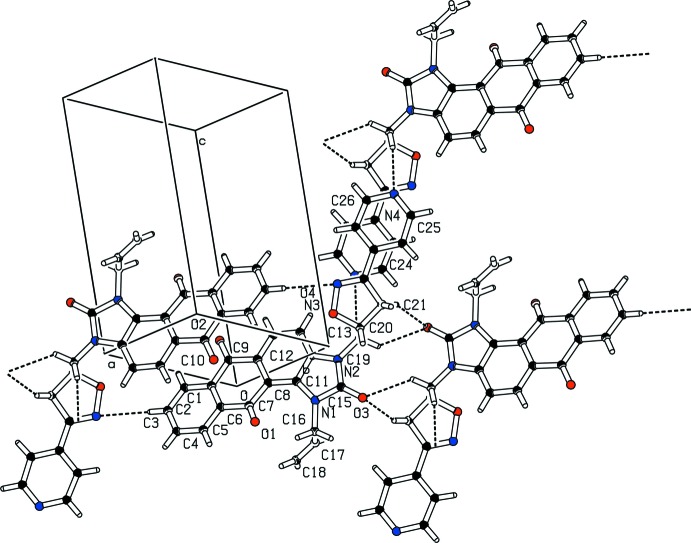
: Intermolecular interactions in the title compound. Hydrogen bonds are shown as dashed lines. Symmetry codes: (i) -x + 1, -y + 1, -z + 2; (ii) -x + 3, -y, -z + 2.

## supplementary crystallographic information

### Comment

Anthraquinone is a versatile organic redox mediator and is useful for applications such as selective H_2_O_2_ production through oxygen reduction reaction (Campos-Martin *et al.*, 2006); photo-cleavage of DNA (Henderson *et al.*, 1998); anticancer activity (anthraquinone as central building block) (Barasch *et al.*, 1999) and enzyme/mediator (Dou *et al.*, 2009) *etc*. Similarly, electrodes that were chemically modified with anthraquinone were useful for several electrochemical (Harish *et al.*, 2009); electrocatalytic (Jürmann *et al.*, 2007; Manisankar *et al.*, 2005); and pH sensor applications (Wong *et al.*, 2004; Lafitte *et al.*, 2008; Wildgoose *et al.*, 2003).

In this work, we aim to prepare new derivatives of anthra [1,2-*d*]imidazole- 2,6,11–trione for biological activities. In a previous study we have synthesized the 1,3-diallyl-1*H*-anthra [1,2-*d*] imidazole-2,6,11(3*H*)-trione (Afrakssou *et al.*, 2010), and, here we have focused in the reactivity of the exocyclic C=C bond of the allyl substituent towards nitriloxides (Afrakssou *et al.*, 2011). The latter are produced as intermediates in the dehydrohalogenation of (*E*)-isonicotinaldehyde oxime by a solution of sodium hypochlorite. The oxime then reacts with 1, 3-diallyl-1*H*-anthra [1, 2 - d] imidazole-2, 6, 11(3*H*)-trione in a biphasic medium (water-chloroform) at 273 K during 4 h to a unique cycloadduct 1-allyl-3-((3-(pyridin-4-yl)-4,5-dihydroisoxazol-5-yl)methyl)- 1*H*-anthra[1,2-*d*]imidazole-2,6,11(3*H*)-trione (Scheme 1).

The molecule of title compound, C_27_H_20_N_4_O_4_, contains four fused rings, three are six-membered rings and one is the five-membered imidazole ring. The imidazole ring is on one side attached to the allyl chain and on the other side to a (3-(pyridin-4-yl)-4,5-dihydroisoxazol-5-yl)methyl group (Fig.1). The fused ring system is almost planar with the largest deviation from the mean plane being 0.232 (2) A° at C7. In this system, the two pairs of adjacent rings are slightly folded along the common edge (C8–C9) making a dihedral angle of 11.17 (6) °. The isoxazole (N3-O4-C20-C21-C22) ring makes dihedral angles of 27.2 (2) ° and 12.7 (2) ° with the imidazole (N1-N2-C11-C12-C15) and pyridine (N4-C23 to C27) rings, respectively.

Weak intermolecular C19–H19A···O3, C21–H21A···O3, C3–H3···O4 and C3–H3···N3 hydrogen bondings ensures the cohesion of the crystal structure as shown in Fig. 2 and Table 2.

### Experimental

To a solution of 1,3-diallyl-1*H*-anthra[1,2-*d*]imidazole-2,6,11(3*H*) -trione (0.5 g, 1.45 mmol) and (*E*)-isonicotinaldehyde oxime (0.44 g, 3.62 mmol) in chloroform (16 ml) was added dropwise a 24% sodium hypochlorite solution (8 ml) at 273 K. Stirring was continued for 4 h. The organic layer was dried over Na_2_SO_4_ and the solvent was evaporated under reduced pressure. The residue was then purified by column chromatography on silica gel using a mixture of hexane/ethyl acetate (1/1) as eluent. The yield of the reaction is of 51%. Orange crystals were isolated after the solvent was allowed to evaporate.

### Refinement

All H atoms could be located in a difference Fourier map. However, they were placed in calculated positions with C—H = 0.93 Å (aromatic and methyne), and C—H = 0.97 Å (methylene) and refined as riding on their parent atoms with *U*_iso_(H) = 1.2 *U*_eq_ (C).

### Crystal data

**Table:**

C_27_H_20_N_4_O_4_	*Z* = 2
*M**_r_* = 464.47	*F*(000) = 484
Triclinic, *P*1	*D*_x_ = 1.403 Mg m^−^^3^
Hall symbol: -P 1	Melting point: 443 K
*a* = 8.0930 (2) Å	Mo *K*α radiation, λ = 0.71073 Å
*b* = 12.1191 (3) Å	Cell parameters from 8485 reflections
*c* = 12.2743 (2) Å	θ = 2.8–26.0°
α = 87.109 (1)°	µ = 0.10 mm^−^^1^
β = 73.612 (1)°	*T* = 296 K
γ = 72.283 (1)°	Block, orange
*V* = 1099.35 (4) Å^3^	0.14 × 0.10 × 0.08 mm

### Data collection

**Table:**

Bruker APEXII CCD diffractometer	3900 reflections with *I* > 2σ(*I*)
Radiation source: microfocus source	*R*_int_ = 0.031
Graphite monochromator	θ_max_ = 28.7°, θ_min_ = 1.7°
φ and ω scans	*h* = −10→10
36799 measured reflections	*k* = −16→16
5659 independent reflections	*l* = −16→16

### Refinement

**Table:**

Refinement on *F*^2^	Primary atom site location: structure-invariant direct methods
Least-squares matrix: full	Secondary atom site location: difference Fourier map
*R*[*F*^2^ > 2σ(*F*^2^)] = 0.048	Hydrogen site location: difference Fourier map
*wR*(*F*^2^) = 0.148	H-atom parameters constrained
*S* = 1.02	*w* = 1/[σ^2^(*F*_o_^2^) + (0.0711*P*)^2^ + 0.2464*P*] where *P* = (*F*_o_^2^ + 2*F*_c_^2^)/3
5659 reflections	(Δ/σ)_max_ < 0.001
316 parameters	Δρ_max_ = 0.37 e Å^−^^3^
0 restraints	Δρ_min_ = −0.20 e Å^−^^3^

### Special details

**Table:**

Geometry. All s.u.'s (except the s.u. in the dihedral angle between two l.s. planes) are estimated using the full covariance matrix. The cell s.u.'s are taken into account individually in the estimation of s.u.'s in distances, angles and torsion angles; correlations between s.u.'s in cell parameters are only used when they are defined by crystal symmetry. An approximate (isotropic) treatment of cell s.u.'s is used for estimating s.u.'s involving l.s. planes.
Refinement. Refinement of *F*^2^ against all reflections. The weighted *R*-factor *wR* and goodness of fit *S* are based on *F*^2^, conventional *R*-factors *R* are based on *F*, with *F* set to zero for negative *F*^2^. The threshold expression of *F*^2^ > σ(*F*^2^) is used only for calculating *R*-factors(gt) *etc*. and is not relevant to the choice of reflections for refinement. *R*-factors based on *F*^2^ are statistically about twice as large as those based on *F*, and *R*- factors based on all data will be even larger.

### Fractional atomic coordinates and isotropic or equivalent isotropic displacement parameters (Å^2^)

**Table:**

	*x*	*y*	*z*	*U*_iso_*/*U*_eq_	
C1	0.5114 (2)	0.62606 (14)	1.11854 (16)	0.0495 (4)	
C2	0.3824 (3)	0.73461 (16)	1.1261 (2)	0.0637 (5)	
H2	0.3864	0.7808	1.0631	0.076*	
C3	0.2504 (3)	0.77342 (18)	1.2256 (2)	0.0742 (7)	
H3	0.1658	0.8462	1.2302	0.089*	
C4	0.2421 (3)	0.70528 (19)	1.3189 (2)	0.0739 (6)	
H4	0.1509	0.7316	1.3860	0.089*	
C5	0.3689 (3)	0.59784 (17)	1.31336 (19)	0.0637 (5)	
H5	0.3630	0.5521	1.3766	0.076*	
C6	0.5051 (2)	0.55819 (14)	1.21343 (15)	0.0488 (4)	
C7	0.6385 (2)	0.44158 (14)	1.20925 (14)	0.0463 (4)	
C8	0.7922 (2)	0.40617 (13)	1.10555 (13)	0.0383 (3)	
C9	0.7841 (2)	0.46768 (13)	1.00512 (13)	0.0417 (3)	
C10	0.6459 (2)	0.58148 (15)	1.00850 (16)	0.0498 (4)	
C11	0.93761 (19)	0.30551 (12)	1.09642 (12)	0.0362 (3)	
C12	1.0529 (2)	0.26122 (13)	0.98806 (12)	0.0376 (3)	
C13	1.0379 (2)	0.31860 (15)	0.89005 (13)	0.0464 (4)	
H13	1.1144	0.2877	0.8194	0.056*	
C14	0.9049 (2)	0.42393 (15)	0.90068 (14)	0.0478 (4)	
H14	0.8960	0.4666	0.8363	0.057*	
C15	1.1529 (2)	0.14078 (13)	1.11712 (13)	0.0382 (3)	
C16	0.9718 (2)	0.24952 (15)	1.29631 (13)	0.0481 (4)	
H16A	1.0353	0.1796	1.3274	0.058*	
H16B	0.8438	0.2670	1.3348	0.058*	
C17	1.0362 (3)	0.34735 (19)	1.31752 (16)	0.0659 (5)	
H17	1.1552	0.3440	1.2807	0.079*	
C18	0.9359 (5)	0.4370 (2)	1.3844 (2)	0.1064 (10)	
H18A	0.8164	0.4428	1.4223	0.128*	
H18B	0.9836	0.4953	1.3942	0.128*	
C19	1.3115 (2)	0.07767 (14)	0.91585 (13)	0.0418 (4)	
H19A	1.4231	0.0492	0.9374	0.050*	
H19B	1.3360	0.1169	0.8450	0.050*	
C20	1.2483 (2)	−0.02358 (14)	0.89840 (13)	0.0426 (4)	
H20	1.2199	−0.0630	0.9698	0.051*	
C21	1.3846 (2)	−0.10974 (14)	0.80505 (14)	0.0473 (4)	
H21A	1.5044	−0.1010	0.7885	0.057*	
H21B	1.3904	−0.1891	0.8245	0.057*	
C22	1.3037 (2)	−0.07338 (14)	0.70869 (13)	0.0433 (4)	
C23	1.3916 (2)	−0.11054 (15)	0.58912 (14)	0.0464 (4)	
C24	1.5579 (3)	−0.19406 (17)	0.55610 (16)	0.0622 (5)	
H24	1.6145	−0.2309	0.6101	0.075*	
C25	1.6397 (3)	−0.2224 (2)	0.44121 (19)	0.0754 (6)	
H25	1.7517	−0.2789	0.4207	0.090*	
C26	1.4083 (4)	−0.0959 (2)	0.39166 (17)	0.0716 (6)	
H26	1.3550	−0.0618	0.3354	0.086*	
C27	1.3145 (3)	−0.06123 (18)	0.50314 (15)	0.0581 (5)	
H27	1.2015	−0.0057	0.5206	0.070*	
N1	1.00212 (17)	0.22965 (11)	1.17475 (10)	0.0385 (3)	
N2	1.17735 (17)	0.15961 (11)	1.00326 (10)	0.0391 (3)	
N3	1.14308 (19)	−0.00366 (13)	0.73868 (12)	0.0502 (4)	
N4	1.5695 (3)	−0.17468 (18)	0.35938 (15)	0.0773 (6)	
O1	0.61239 (19)	0.37492 (12)	1.28523 (12)	0.0702 (4)	
O2	0.6424 (2)	0.63732 (13)	0.92318 (13)	0.0750 (4)	
O3	1.24659 (15)	0.06244 (10)	1.15952 (9)	0.0478 (3)	
O4	1.08962 (15)	0.01876 (11)	0.85700 (10)	0.0524 (3)	

### Atomic displacement parameters (Å^2^)

**Table:**

	*U*^11^	*U*^22^	*U*^33^	*U*^12^	*U*^13^	*U*^23^
C1	0.0437 (9)	0.0358 (8)	0.0712 (11)	−0.0073 (7)	−0.0243 (8)	0.0000 (8)
C2	0.0596 (12)	0.0400 (10)	0.0947 (15)	−0.0030 (8)	−0.0391 (11)	0.0001 (10)
C3	0.0565 (12)	0.0461 (11)	0.1142 (19)	0.0086 (9)	−0.0374 (12)	−0.0199 (12)
C4	0.0505 (11)	0.0595 (13)	0.0962 (17)	0.0019 (9)	−0.0117 (11)	−0.0244 (12)
C5	0.0499 (10)	0.0529 (11)	0.0731 (13)	−0.0035 (8)	−0.0046 (9)	−0.0098 (9)
C6	0.0404 (8)	0.0387 (9)	0.0618 (10)	−0.0068 (7)	−0.0107 (7)	−0.0039 (7)
C7	0.0421 (8)	0.0378 (8)	0.0510 (9)	−0.0075 (7)	−0.0056 (7)	0.0013 (7)
C8	0.0387 (8)	0.0332 (7)	0.0417 (8)	−0.0095 (6)	−0.0105 (6)	0.0011 (6)
C9	0.0428 (8)	0.0371 (8)	0.0479 (9)	−0.0112 (6)	−0.0186 (7)	0.0064 (6)
C10	0.0497 (9)	0.0415 (9)	0.0625 (11)	−0.0120 (7)	−0.0259 (8)	0.0115 (8)
C11	0.0379 (7)	0.0337 (7)	0.0350 (7)	−0.0096 (6)	−0.0086 (6)	0.0019 (6)
C12	0.0368 (7)	0.0378 (8)	0.0374 (7)	−0.0095 (6)	−0.0107 (6)	−0.0015 (6)
C13	0.0504 (9)	0.0511 (10)	0.0351 (8)	−0.0126 (8)	−0.0109 (7)	0.0002 (7)
C14	0.0559 (10)	0.0498 (10)	0.0400 (8)	−0.0161 (8)	−0.0184 (7)	0.0104 (7)
C15	0.0361 (7)	0.0371 (8)	0.0392 (8)	−0.0081 (6)	−0.0102 (6)	−0.0004 (6)
C16	0.0589 (10)	0.0446 (9)	0.0329 (8)	−0.0097 (8)	−0.0073 (7)	0.0039 (6)
C17	0.0908 (15)	0.0682 (13)	0.0444 (10)	−0.0304 (12)	−0.0208 (10)	0.0021 (9)
C18	0.173 (3)	0.0811 (18)	0.0647 (15)	−0.0454 (19)	−0.0226 (17)	−0.0155 (13)
C19	0.0333 (7)	0.0453 (9)	0.0395 (8)	−0.0044 (6)	−0.0055 (6)	−0.0059 (6)
C20	0.0400 (8)	0.0430 (8)	0.0386 (8)	−0.0065 (6)	−0.0079 (6)	−0.0011 (6)
C21	0.0465 (9)	0.0412 (9)	0.0468 (9)	−0.0034 (7)	−0.0109 (7)	−0.0057 (7)
C22	0.0423 (8)	0.0434 (9)	0.0439 (8)	−0.0153 (7)	−0.0083 (7)	−0.0019 (7)
C23	0.0532 (9)	0.0459 (9)	0.0426 (8)	−0.0237 (8)	−0.0075 (7)	−0.0013 (7)
C24	0.0672 (12)	0.0572 (11)	0.0503 (10)	−0.0154 (9)	−0.0008 (9)	−0.0049 (8)
C25	0.0807 (15)	0.0671 (14)	0.0613 (13)	−0.0206 (12)	0.0070 (11)	−0.0112 (11)
C26	0.1029 (18)	0.0818 (15)	0.0465 (10)	−0.0523 (15)	−0.0207 (11)	0.0064 (10)
C27	0.0685 (12)	0.0629 (12)	0.0505 (10)	−0.0312 (10)	−0.0166 (9)	0.0033 (9)
N1	0.0419 (7)	0.0348 (6)	0.0330 (6)	−0.0055 (5)	−0.0083 (5)	0.0013 (5)
N2	0.0373 (6)	0.0388 (7)	0.0349 (6)	−0.0040 (5)	−0.0078 (5)	−0.0020 (5)
N3	0.0441 (8)	0.0569 (9)	0.0486 (8)	−0.0131 (7)	−0.0127 (6)	−0.0051 (7)
N4	0.1033 (16)	0.0782 (13)	0.0495 (10)	−0.0441 (12)	−0.0005 (10)	−0.0073 (9)
O1	0.0647 (8)	0.0511 (8)	0.0652 (8)	−0.0048 (6)	0.0122 (6)	0.0122 (6)
O2	0.0784 (10)	0.0606 (9)	0.0739 (9)	−0.0016 (7)	−0.0277 (8)	0.0263 (7)
O3	0.0435 (6)	0.0452 (6)	0.0494 (6)	−0.0029 (5)	−0.0168 (5)	0.0054 (5)
O4	0.0367 (6)	0.0643 (8)	0.0502 (7)	−0.0081 (5)	−0.0083 (5)	−0.0125 (6)

### Geometric parameters (Å, º)

**Table:**

C1—C6	1.390 (3)	C16—C17	1.493 (3)
C1—C2	1.397 (2)	C16—H16A	0.9700
C1—C10	1.479 (3)	C16—H16B	0.9700
C2—C3	1.368 (3)	C17—C18	1.300 (3)
C2—H2	0.9300	C17—H17	0.9300
C3—C4	1.376 (3)	C18—H18A	0.9300
C3—H3	0.9300	C18—H18B	0.9300
C4—C5	1.382 (3)	C19—N2	1.4525 (18)
C4—H4	0.9300	C19—C20	1.511 (2)
C5—C6	1.387 (2)	C19—H19A	0.9700
C5—H5	0.9300	C19—H19B	0.9700
C6—C7	1.487 (2)	C20—O4	1.453 (2)
C7—O1	1.218 (2)	C20—C21	1.525 (2)
C7—C8	1.479 (2)	C20—H20	0.9800
C8—C11	1.397 (2)	C21—C22	1.494 (2)
C8—C9	1.415 (2)	C21—H21A	0.9700
C9—C14	1.388 (2)	C21—H21B	0.9700
C9—C10	1.482 (2)	C22—N3	1.278 (2)
C10—O2	1.220 (2)	C22—C23	1.465 (2)
C11—N1	1.3943 (19)	C23—C24	1.380 (3)
C11—C12	1.412 (2)	C23—C27	1.393 (3)
C12—C13	1.376 (2)	C24—C25	1.389 (3)
C12—N2	1.3760 (19)	C24—H24	0.9300
C13—C14	1.381 (2)	C25—N4	1.315 (3)
C13—H13	0.9300	C25—H25	0.9300
C14—H14	0.9300	C26—N4	1.326 (3)
C15—O3	1.2171 (18)	C26—C27	1.380 (3)
C15—N2	1.3726 (19)	C26—H26	0.9300
C15—N1	1.3927 (19)	C27—H27	0.9300
C16—N1	1.4621 (19)	N3—O4	1.4084 (18)
			
C6—C1—C2	119.35 (18)	C18—C17—C16	123.9 (2)
C6—C1—C10	120.62 (15)	C18—C17—H17	118.1
C2—C1—C10	119.92 (17)	C16—C17—H17	118.1
C3—C2—C1	120.4 (2)	C17—C18—H18A	120.0
C3—C2—H2	119.8	C17—C18—H18B	120.0
C1—C2—H2	119.8	H18A—C18—H18B	120.0
C2—C3—C4	120.29 (19)	N2—C19—C20	111.63 (12)
C2—C3—H3	119.9	N2—C19—H19A	109.3
C4—C3—H3	119.9	C20—C19—H19A	109.3
C3—C4—C5	120.2 (2)	N2—C19—H19B	109.3
C3—C4—H4	119.9	C20—C19—H19B	109.3
C5—C4—H4	119.9	H19A—C19—H19B	108.0
C4—C5—C6	120.1 (2)	O4—C20—C19	108.93 (13)
C4—C5—H5	120.0	O4—C20—C21	104.00 (12)
C6—C5—H5	120.0	C19—C20—C21	112.82 (13)
C5—C6—C1	119.71 (17)	O4—C20—H20	110.3
C5—C6—C7	118.96 (17)	C19—C20—H20	110.3
C1—C6—C7	121.30 (15)	C21—C20—H20	110.3
O1—C7—C8	121.68 (15)	C22—C21—C20	100.34 (13)
O1—C7—C6	120.30 (15)	C22—C21—H21A	111.7
C8—C7—C6	117.76 (14)	C20—C21—H21A	111.7
C11—C8—C9	116.86 (13)	C22—C21—H21B	111.7
C11—C8—C7	123.26 (14)	C20—C21—H21B	111.7
C9—C8—C7	119.39 (14)	H21A—C21—H21B	109.5
C14—C9—C8	121.33 (15)	N3—C22—C23	120.26 (16)
C14—C9—C10	117.88 (15)	N3—C22—C21	113.77 (14)
C8—C9—C10	120.79 (15)	C23—C22—C21	125.98 (15)
O2—C10—C1	120.60 (16)	C24—C23—C27	116.82 (17)
O2—C10—C9	121.25 (17)	C24—C23—C22	121.28 (17)
C1—C10—C9	118.15 (15)	C27—C23—C22	121.87 (17)
N1—C11—C8	134.04 (13)	C23—C24—C25	119.1 (2)
N1—C11—C12	106.27 (12)	C23—C24—H24	120.4
C8—C11—C12	119.68 (13)	C25—C24—H24	120.4
C13—C12—N2	129.78 (14)	N4—C25—C24	124.4 (2)
C13—C12—C11	122.57 (14)	N4—C25—H25	117.8
N2—C12—C11	107.59 (13)	C24—C25—H25	117.8
C12—C13—C14	117.41 (14)	N4—C26—C27	124.4 (2)
C12—C13—H13	121.3	N4—C26—H26	117.8
C14—C13—H13	121.3	C27—C26—H26	117.8
C13—C14—C9	121.59 (15)	C26—C27—C23	119.0 (2)
C13—C14—H14	119.2	C26—C27—H27	120.5
C9—C14—H14	119.2	C23—C27—H27	120.5
O3—C15—N2	126.58 (14)	C15—N1—C11	109.31 (12)
O3—C15—N1	126.66 (14)	C15—N1—C16	118.49 (13)
N2—C15—N1	106.75 (13)	C11—N1—C16	129.16 (13)
N1—C16—C17	111.20 (13)	C15—N2—C12	109.96 (12)
N1—C16—H16A	109.4	C15—N2—C19	122.55 (13)
C17—C16—H16A	109.4	C12—N2—C19	127.42 (13)
N1—C16—H16B	109.4	C22—N3—O4	109.39 (13)
C17—C16—H16B	109.4	C25—N4—C26	116.19 (19)
H16A—C16—H16B	108.0	N3—O4—C20	108.52 (11)
			
C6—C1—C2—C3	−0.5 (3)	N1—C16—C17—C18	125.5 (2)
C10—C1—C2—C3	175.79 (17)	N2—C19—C20—O4	63.70 (16)
C1—C2—C3—C4	−0.7 (3)	N2—C19—C20—C21	178.64 (13)
C2—C3—C4—C5	1.0 (3)	O4—C20—C21—C22	18.17 (16)
C3—C4—C5—C6	−0.1 (3)	C19—C20—C21—C22	−99.71 (15)
C4—C5—C6—C1	−1.1 (3)	C20—C21—C22—N3	−12.02 (19)
C4—C5—C6—C7	−179.20 (18)	C20—C21—C22—C23	168.07 (15)
C2—C1—C6—C5	1.4 (3)	N3—C22—C23—C24	−173.79 (16)
C10—C1—C6—C5	−174.83 (17)	C21—C22—C23—C24	6.1 (3)
C2—C1—C6—C7	179.42 (16)	N3—C22—C23—C27	8.2 (3)
C10—C1—C6—C7	3.2 (3)	C21—C22—C23—C27	−171.93 (16)
C5—C6—C7—O1	11.5 (3)	C27—C23—C24—C25	1.2 (3)
C1—C6—C7—O1	−166.50 (18)	C22—C23—C24—C25	−176.92 (17)
C5—C6—C7—C8	−174.24 (16)	C23—C24—C25—N4	0.0 (3)
C1—C6—C7—C8	7.7 (2)	N4—C26—C27—C23	0.3 (3)
O1—C7—C8—C11	−13.9 (3)	C24—C23—C27—C26	−1.4 (3)
C6—C7—C8—C11	171.97 (15)	C22—C23—C27—C26	176.75 (16)
O1—C7—C8—C9	157.86 (17)	O3—C15—N1—C11	176.95 (15)
C6—C7—C8—C9	−16.3 (2)	N2—C15—N1—C11	−2.11 (16)
C11—C8—C9—C14	6.1 (2)	O3—C15—N1—C16	14.9 (2)
C7—C8—C9—C14	−166.19 (15)	N2—C15—N1—C16	−164.21 (13)
C11—C8—C9—C10	−173.72 (14)	C8—C11—N1—C15	178.90 (16)
C7—C8—C9—C10	14.0 (2)	C12—C11—N1—C15	−0.01 (16)
C6—C1—C10—O2	174.24 (18)	C8—C11—N1—C16	−21.5 (3)
C2—C1—C10—O2	−2.0 (3)	C12—C11—N1—C16	159.59 (15)
C6—C1—C10—C9	−5.8 (2)	C17—C16—N1—C15	99.02 (18)
C2—C1—C10—C9	178.01 (15)	C17—C16—N1—C11	−59.0 (2)
C14—C9—C10—O2	−2.8 (3)	O3—C15—N2—C12	−175.56 (15)
C8—C9—C10—O2	176.99 (16)	N1—C15—N2—C12	3.51 (17)
C14—C9—C10—C1	177.19 (15)	O3—C15—N2—C19	7.3 (2)
C8—C9—C10—C1	−3.0 (2)	N1—C15—N2—C19	−173.60 (13)
C9—C8—C11—N1	172.45 (16)	C13—C12—N2—C15	173.53 (16)
C7—C8—C11—N1	−15.6 (3)	C11—C12—N2—C15	−3.55 (17)
C9—C8—C11—C12	−8.8 (2)	C13—C12—N2—C19	−9.5 (3)
C7—C8—C11—C12	163.18 (14)	C11—C12—N2—C19	173.38 (13)
N1—C11—C12—C13	−175.20 (14)	C20—C19—N2—C15	78.33 (18)
C8—C11—C12—C13	5.7 (2)	C20—C19—N2—C12	−98.24 (18)
N1—C11—C12—N2	2.14 (16)	C23—C22—N3—O4	−179.88 (13)
C8—C11—C12—N2	−176.96 (13)	C21—C22—N3—O4	0.2 (2)
N2—C12—C13—C14	−176.07 (16)	C24—C25—N4—C26	−1.1 (3)
C11—C12—C13—C14	0.6 (2)	C27—C26—N4—C25	1.0 (3)
C12—C13—C14—C9	−3.5 (3)	C22—N3—O4—C20	12.78 (18)
C8—C9—C14—C13	0.1 (3)	C19—C20—O4—N3	100.89 (14)
C10—C9—C14—C13	179.91 (15)	C21—C20—O4—N3	−19.64 (16)

### Hydrogen-bond geometry (Å, º)

**Table:**

*D*—H···*A*	*D*—H	H···*A*	*D*···*A*	*D*—H···*A*
C3—H3···N3^i^	0.93	2.58	3.471 (2)	160
C3—H3···O4^i^	0.93	2.67	3.470 (2)	145
C19—H19*A*···O3^ii^	0.97	2.56	3.3356 (19)	137
C21—H21*A*···O3^ii^	0.97	2.45	3.350 (2)	154

Symmetry codes: (i) −*x*+1, −*y*+1, −*z*+2; (ii) −*x*+3, −*y*, −*z*+2.
